# *In vivo* Molecular Imaging and Radionuclide (^131^I) Therapy of Human Nasopharyngeal Carcinoma Cells Transfected with a Lentivirus Expressing Sodium Iodide Symporter

**DOI:** 10.1371/journal.pone.0116531

**Published:** 2015-01-26

**Authors:** Shuo Shi, Min Zhang, Rui Guo, Ying Miao, Jiajia Hu, Yun Xi, Biao Li

**Affiliations:** Department of Nuclear Medicine, Shanghai Jiaotong University School of Medicine, Shanghai, China; University of Naples Federico II, ITALY

## Abstract

**Introduction:**

Despite recent improvements in the survival rates for nasopharyngeal carcinoma (NPC), novel treatment strategies are required to improve distant metastasis-free survival. The sodium iodine symporter (NIS) gene has been applied for *in vivo* imaging and cancer therapy. In this study, we examined the potential of NIS gene therapy as a therapeutic approach in NPC by performing non-invasive imaging using ^125^I and ^131^I therapy *in vivo*.

**Methods:**

We constructed a lentiviral vector expressing NIS and enhanced green fluorescent protein (EGFP) under the control of the human elongation factor-1α (EF1α) promoter, and stably transfected the vector into CNE-2Z NPC cells to create CNE-2Z-NIS cells. CNE-2Z and CNE-2Z-NIS tumor xenografts were established in nude mice; ^125^I uptake, accumulation and efflux were measured using micro-SPECT/CT imaging; the therapeutic effects of treatment with ^131^I were assessed over 25 days by measuring tumor volume and immunohistochemical staining of the excised tumors.

**Results:**

qPCR, immunofluorescence and Western blotting confirmed that CNE-2Z-NIS cells expressed high levels of NIS mRNA and protein. CNE-2Z-NIS cells and xenografts took up and accumulated significantly more ^125^I than CNE-2Z cells and xenografts. *In vitro*, ^131^I significantly reduced the clonogenic survival of CNE-2Z-NIS cells. *In vivo*, ^131^I effectively inhibited the growth of CNE-2Z-NIS xenografts. At the end of ^131^I therapy, CNE-2Z-NIS xenograft tumor cells expressed higher levels of NIS and caspase-3 and lower levels of Ki-67.

**Conclusion:**

Lentiviruses effectively delivered and mediated long-lasting expression of NIS in CNE-2Z cells which enabled uptake and accumulation of radioisotopes and provided a significant therapeutic effect in an *in vivo* model of NPC. NIS-mediated radioiodine treatment merits further investigation as a potentially effective, low toxicity therapeutic strategy for NPC.

## Introduction

Nasopharyngeal carcinoma (NPC) represents only a small proportion of head and neck cancers worldwide, but has a high rate of incidence in southern China, Southeast Asia, northern Africa and Alaska [1]. Radiotherapy is the mainstream treatment for primary NPC. Due to improved radiotherapy techniques and chemotherapy strategies, the 5-year survival rate for NPC has increased from 50% in the 1980s to 80% at present [2]. However, 15%–58% of patients still experience recurrent disease, most of whom recur within 3 years of initial treatment [3]. Therefore, novel efficient therapies need to be explored to improve the management of recurrent, residual and metastatic NPC [4].

Sodium iodide symporter (NIS) is an intrinsic membrane glycoprotein responsible for iodide transport. NIS is expressed in thyroid follicular cells and others cells containing a lactoperoxidase system [5, 6]. It has been targeted as a safe and effective approach for the imaging and treatment of thyroid disease [7]. A number of other studies have successfully introduced ectopic expression of NIS for imaging and therapy in non-thyroidal carcinoma [8, 9]. The field of gene therapy has made considerable advances in the last decade due to the development of new vector systems, including lentiviral vectors, and an increasing repertoire of therapeutic genes [10]. Thus, *NIS*-based gene therapy may potentially have therapeutic value in other intractable malignancies.

In the current study, we employed a lentiviral vector to express a functional *NIS* gene in NPC cells. The potential of *NIS* as an imaging reporter gene for the uptake and accumulation of ^125^I, and target gene for ^131^I therapy were investigated *in vitro* and *in vivo* using a xenograft model of NPC.

## Materials and Methods

### Virus production and cell culture

Lv-EF1α-OCT_4_-IRES-EGFP was kindly provided by the Institute of Molecular Biology, Chinese Academy of Sciences; pcDNA3.1-NIS was obtained from our own library [11]. The *NIS* gene was amplified from pcDNA3.1-NIS by PCR using the primers: forward (5’-GCGCGGATCCCGGGTATCGATGGAGGCCGTG-3’) and reverse (5’-CGCGTCTAGATCAGAGGTTTGTAGGTAGTGAGC-3’), digested with *Xba*I and *BamH*I, and cloned into the *Xba*I and *BamH*I sites of Lv-EF1α-OCT_4_-IRES-EGFP generating a functional vector featuring *NIS* under the control of the human elongation factor-1α (EF1α) promoter (the *OCT_4_* transgene of Lv-EF1α-OCT_4_-IRES-EGFP was replaced with *NIS*).

HEK293T cell line (Cell Bank of the Chinese Academy of Science, Shanghai, China) was cultured in RPMI-1640 medium supplemented with 10% FBS(Fetal Bovine Serum) and 1% penicillin/streptomycin.

Virus particles were generated by cotransfection of HEK293T cells with Lv-EF1α-NIS-IRES-EGFP and the three packaging plasmids pRsv-REV, pMDIg-pRRE and pMD2G(Biovector Science Lab, Beijing, China). The virus particles were harvested by collecting the cell culture medium at 48 h post-transfection; the supernatants were filtered through 0.45 µm filters, centrifuged at 10,000 *g* for 15 min and the resulting pellet was resuspended in 100 μl culture medium.

### CNE-2Z cell culture

The human NPC cell line CNE-2Z (Cell Bank of the Chinese Academy of Science, Shanghai, China) was cultured in RPMI-1640 medium supplemented with 10% FBS (Fetal Bovine Serum) and 1% penicillin/streptomycin. CNE-2Z cells were infected overnight with Lv-EF1α-NIS-IRES-EGFP at a multiplicity of infection (MOI) of 20. To obtain CNE-2Z cells stably transfected with Lv-EF1α-NIS-IRES-EGFP (CNE-2Z-NIS cells), the EGFP-positive cells were sorted using a FACScan (Becton Dickinson, CA, USA) following the standard procedure with a standard excitation wavelength of 488 nm. The sorted cells were cultured for about 2 weeks, sorted again, the process repeated until the EGFP-positive cells is nearly 100% after cultured 2 weeks the last time sorted.

### qPCR, immunofluorescence and Western blotting

CNE-2Z and CNE-2Z-NIS cells were lysed using TRIzol (Invitrogen, Carlsbad, CA, USA), total RNA was extracted. cDNA was synthesized using the PrimeScript First Strand cDNA Synthesis kit (Takara Bio Inc., Dalian, China). Real Time PCR was performed using SYBR Premix Ex Taq II (Takara Bio Inc.) according to the manufacturer’s instructions. The *NIS* gene was amplified with forward (5’-GTACATTG TAGCCACGATGCTGTA-3’) and reverse primers (5’-CCGTGTAGAAGGTGCAGATAATTC-3’), 95°C for 30 s followed by 40 cycles of 5 s at 95°C and 30 s at 60°C. *GAPDH* was co-amplified using the primers: forward (5’-GTCAAGCTCATTTCCTGGTATGAC-3’) and reverse (5’- CTCTCTCTTCCTCTTGTGCTCTTG -3’). To correct for differences in both quality and quantity between samples, according to the manufacture’s protocol, *NIS* expression level was normalized to that of the *GAPDH* endogenous reference as given by: F value = 2^-ΔΔCt^ [12].

CNE-2Z cells and CNE-2Z-NIS cells were incubated in lysis buffer (99% SDS lysis buffer, 1% PMSF) on ice, centrifuged at 10,000 ×*g* and the protein concentrations of the supernatants were measured using the BCA Protein Assay Kit (Beyotime Inst Biotech, Shanghai, China). Equal quantities of protein were subjected to Western blotting using polyclonal goat anti-NIS antibody (Santa Cruz Biotechnology, CA, USA; 1:500), *GAPDH* (Beyotime Inst Biotech, Shanghai, China; 1:1000), anti-goat IgG-HRP (MultiSciences Biotech Co. Shanghai, China; 1:5,000).

The same polyclonal NIS antibody (1:100) was used for immunofluorescence in combination with DyLight594 rabbit anti-goat IgG (MultiSciences Biotech Co., Shanghai, China; 1:300) and images were observed with a fluorescence microscope (Olympus, Tokyo, Japan)

### Cell viability assay

CNE-2Z and CNE-2Z-NIS cells were plated into 96-well plates (2 × 10^3^ cells/well), incubated for 12, 24, 36,48 or 72 h, the blank group contained only medium without cells. 10 μl CCK-8 reagent (Beyotime Inst. Biotech, Shanghai, China) was added to the wells and the cells were incubated for 1 h. Absorbance values were measured using a Multiskan MK3 microplate reader (Thermo Scientific, Hudson, USA) at 450 nm. The absorbance of cells = (A_test_-A_blank_), A_test_ represents absorbance of each experimental group, and A_blank_ represents absorbance of each blank group. The mean ± standard deviation (SD) values of quadruplicate replicates from at least three independent experiments are presented.

### ^125^I uptake and efflux studies

^125^I uptake and efflux were determined in triplicate as previously described [13]. The day before, CNE-2Z and CNE-2Z-NIS cells were plated (2 × 10^5^ cells/well) in 24-well plates. 24 hours later, 500 μl of Hank’s balanced salt solution (HBSS) containing 3.7 kBq ^125^I and 10 μmol/L sodium iodide (NaI) was added. CNE-2Z-NIS cells in the inhibition group were treated with 50 μM sodium perchlorate (NaClO_4_). The cells were incubated at 37°C for 5–120 min, washed twice with ice-cold HBSS and lysed using 0.5 mol/L NaOH. The radioactivity (counts per minute, cpm) of the cell lysates was measured using an automatic γ counter (Shanghai Rihuan Company, Shanghai, China).

For the efflux studies, CNE-2Z-NIS cells were incubated with 3.7 kBq Na^125^I and 10 μM NaI in 500 μl of HBSS at 37°C for 60 min, washed twice with HBSS, and incubated in 500 μl of HBSS containing 10 μM NaI (without radioactive Na^125^I). After the same treatment above, CNE-2Z-NIS cells in the inhibition group were treated with 50 μM sodium perchlorate (NaClO_4_). Every 5 min (5–40 min), the buffer was replaced and the radioactivity of the solutions was determined. After removal of the last sample (at 40 min), the cells were lysed using 0.5 mol/L NaOH. Total radioactivity at the initiation of the efflux study was calculated by adding final cell radioactivity to total media radioactivity. Na^125^I remaining activity at different time point = (total radioactivity-the sum of radioactivity of the solution at and before the time point) / total radioactivity.

### *In vitro* clonogenic assay

CNE-2Z and CNE-2Z-NIS cells were plated into 10 cm culture dishes (6 × 10^6^ cells/dish) and 4.6 MBq ^131^I in HBSS was added. After 8 h, the cells were washed three times with HBSS, trypsinized and 1,000 cells were plated into each well of 6-well culture plates. On day 7, the cells were stained with 1 ml of Crystal Violet Staining Solution (Beyotime Inst Biotech) for 10 min and colonies containing more than 50 cells were counted; results are expressed as the percentage of surviving cells. The survival rate was expressed as the percentage of colonies to that of the blank group without ^131^I incubation. Data are represented as means ± standard deviation (SD).

### Establishment of xenograft tumors in nude mice

24 male Balb/c–nude mice (4 weeks–old; Shanghai Slaccas Experiment Animal Corporation, Shanghai Institute for Biological Science, China) were subcutaneously injected with 5 × 10^6^ CNE-2Z or CNE-2Z-NIS cells in 150 μl of PBS in each flank. The mice were euthanized by cervical vertebra dislocation at the end of the experiments. The animal studies were approved by the local Ethics Committee (Shanghai Jiao Tong University, School of Medicine) and performed according to ethical principles of animal experimentation.

### Micro-SPECT/CT imaging

The CNE and CNE-2Z-NIS tumor-bearing mice (5 weeks–old) were *i.v.* injected with 10.5 MBq of ^125^I, anesthesia was induced and maintained by isoflurane inhalation, and the mice were placed in a spread-prone position and scanned using a small-animal micro-SPECT scanner (Bioscan, Washington, USA) at 10, 30, 60 min, 2, 4, 8, 24 and 32 h after injection of ^125^I. CT images were acquired (CTDI = 6.1 cGy) before whole-body NanoSPECT images (10 s/frame for systematic scans) were obtained, without moving the mice. The images were processed and reconstructed using Nuclear v1.02 software (Bioscan), HiSPECT 1.4.2 software (Bioscan) for image acquisition and InVivoScope 1.44 software (Bioscan) for image analysis. Regions of interest (ROIs) were drawn around the visible organs and the radioactivity per volume unit (Conc) in the ROIs was measured using InVivoScope 1.44 software (Bioscan).

### *In vivo* ^131^I therapy

10 days prior to *in vivo* therapy, *L*-thyroxine (5 mg/L; L-T4; Merck KGaA, Darmstadt, Germany) was added to the drinking water to maximize radioiodine uptake by the tumors and minimize iodide uptake by the thyroid gland. Water consumption has been monitored carefully in each animal so that each nude mouse can drink almost the same water each day. Treatment was initiated when the tumors reached 3–5 mm in diameter (~70mm^3^). Two groups of CNE-2Z-NIS and CNE-2Z tumor-bearing mice were *i.v.* injected with 37 MBq of ^131^I on day 1 (6 mice/group), other two groups of CNE-2Z-NIS and CNE-2Z tumor-bearing mice were *i.v.* injected with 150μl PBS on day 1 (6 mice/group). Tumor size was measured on day 7 after ^131^I injection and every 3 days thereafter up to day 25 using calipers; tumor volume was calculated using: volume (mm^3^) = (L × W^2^)/ 2 [14].

### Histology and immunohistochemistry

25 days after administration of ^131^I, the animals were sacrificed by cervical vertebra dislocation and the tumors were, cryosectioned (5 μm) and subjected to immunohistochemical analysis using rabbit anti-human NIS antibody (1:50; Proteintech, CHI, USA), rabbit anti-human caspase-3 antibody (1:30; Epitomics, California, USA) and rabbit anti-human Ki67 antibody (1:200; Thermo Scientific, Fremont, USA). Immunohistochemical analysis was analyzed through image pro plus software (Media CY Company). For every section, the integral optical density (IOD) of every visual field was calculated. Data are represented as means ± standard deviation (SD).

### Statistical analysis

Data was analyzed using GaphPad Prism software (version 5.0; GraphPad Software, Inc., San Diego, USA); mean ±SD values are presented. Statistical analyses were performed using two-tailed Student’s *t*-tests for group differences and analysis of variance (ANOVA) for group differences separately. For all analyses, *p* < 0.05 was considered statistically significant.

## Results

### Stable expression of NIS in CNE-2Z-NIS cells

**Figure 1:**
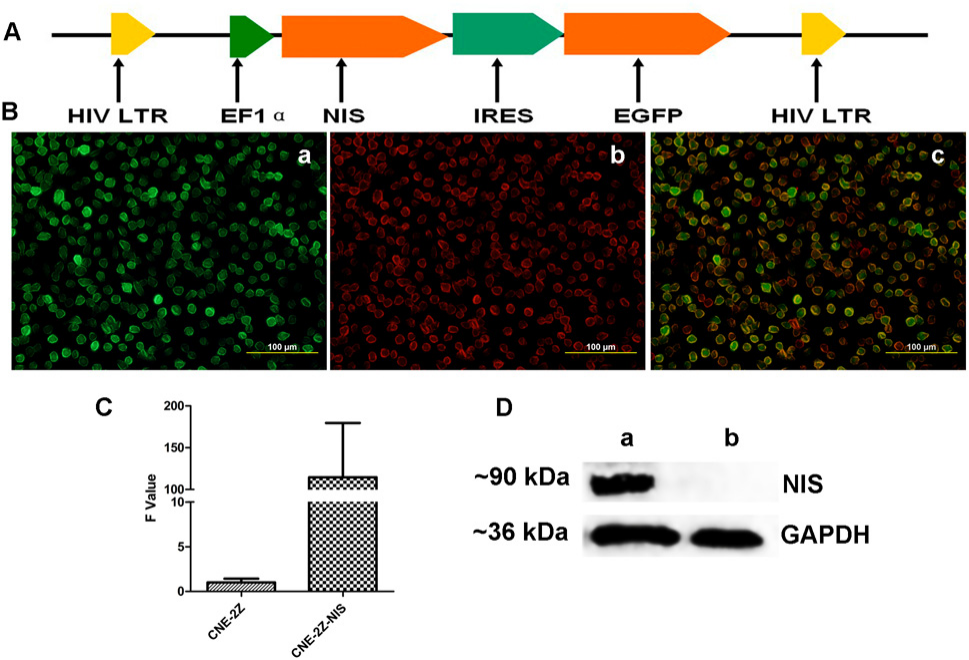
Construction and expression of a two-gene vector containing *NIS* and *EGFP*. (A) Schematic representation of the functional NIS gene and EGFP (green fluorescent protein reporter) gene in Lv-EF1α-NIS-IRES-EGFP. (B) Immunofluorescence showing that NIS and EGFP were strongly expressed in the CNE-2Z-NIS cells. (a: green color shows EGFP expression; b: red color shows NIS expression; c: a merged picture) (magnification, 400×). (C) qPCR analysis of NIS expression in CNE-2Z and CNE-2Z-NIS cells. NIS mRNA expression normalized to the GAPDH endogenous reference was detected by qRT-PCR. Results are expressed as means ± SD of three independent experiments. (D) Western blot analysis of NIS expression in CNE-2Z and CNE-2Z-NIS cells. NIS protein (~90 kDa) expression in CNE-2Z-NIS but not in CNE-2Z cells was analyzed by Western blot. GAPDH (~36 kDa) was used as an internal control (a: CNE-2Z-NIS; b: CNE-2Z). All experiments were performed in triplicate.

The Lv-EF1α-NIS-IRES-EGFP vector was designed to transcribe *NIS* and *EGFP* using an internal ribosomal entry site (IRES). *NIS* was cloned between the EF1α promoter and the IRES (Fig. 1A). Immunofluorescent staining, qPCR and Western blotting confirmed that CNE-2Z cells transfected with Lv-EF1α-NIS-IRES-EGFP (hereafter referred to as CNE-2Z-NIS cells) expressed high levels of NIS mRNA and protein (~90 kDa). (Fig. 1B, C, D).

### CCK-8 assay

**Figure 2:**
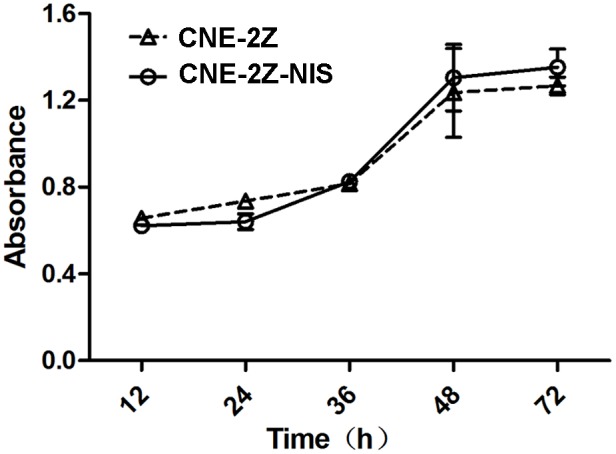
Effect of Lv-EF1α-NIS-IRES-EGFP on the proliferation of CNE-2Z cells. CNE-2Z cells and CNE-2Z-NIS cells were subjected to the CCK-8 assay at 12, 24, 36, 48 and 72 h. Results are means ± SD of three independent experiments.

There was no significant difference in cell viability between CNE-2Z and CNE-2Z-NIS cells (*p*> 0.05) (Fig. 2).

### Uptake and efflux of ^125^I by NPC cells expressing NIS *in vitro*

**Figure 3:**
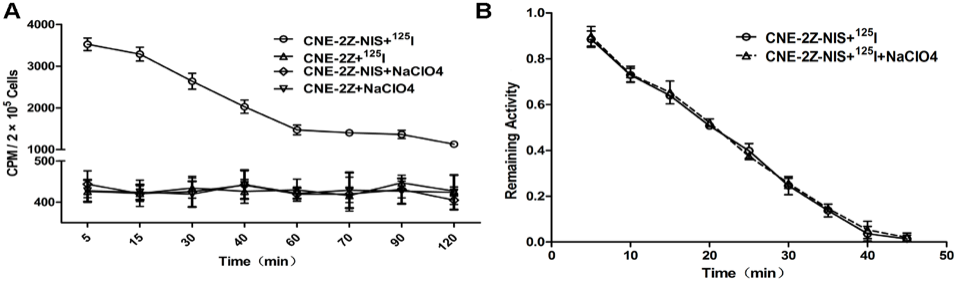
^125^ I uptake and efflux assays. (A) CNE-2Z-NIS and CNE-2Z cells were incubated with Na^125^I at 37°C for 5–120 min. In the inhibition group, CNE-2Z-NIS and CNE-2Z cells were incubated with ^125^I + 50 μM sodium perchlorate. Results are expressed as means ± SD of three independent experiments. (B) Iodine was rapidly effluxed from CNE-2Z-NIS cells and CNE-2Z-NIS cells treated with 50 μM sodium perchlorate, with a *t*_1/2_ of approximately 20 min.

The functional activity of NIS protein was clearly shown by its cellular iodide uptake. ^125^I uptake by CNE-2Z-NIS cells varied depending on the incubation time. After ^125^I added in CNE-2Z-NIS cells, ^125^I was immediately absorbed by NIS protein, and peaked at approximately 3,500 cpm at 5 min; this was 8-fold higher than the level of ^125^I uptake by CNE-2Z cells at the same time point. There was no functional iodide uptake observed in the CNE-2Z cells. ^125^I uptake by CNE-2Z-NIS cells could be completely blocked by sodium perchlorate (Fig. 3A). Because the absorbed iodide can’t be synthesized as the organic iodide polymer, neither can it be stored in CNE-2Z-NIS cells like in thyroid cells, it was rapidly effluxed from CNE-2Z-NIS cells and CNE-2Z-NIS cells treated with 50 μM sodium perchlorate (NaClO_4_), with a same t_1/2_ of approximately 20 min (Fig. 3B).

### *In vivo* imaging of the biodistribution of ^125^I in mice bearing NPC xenografts expressing NIS

**Figure 4:**
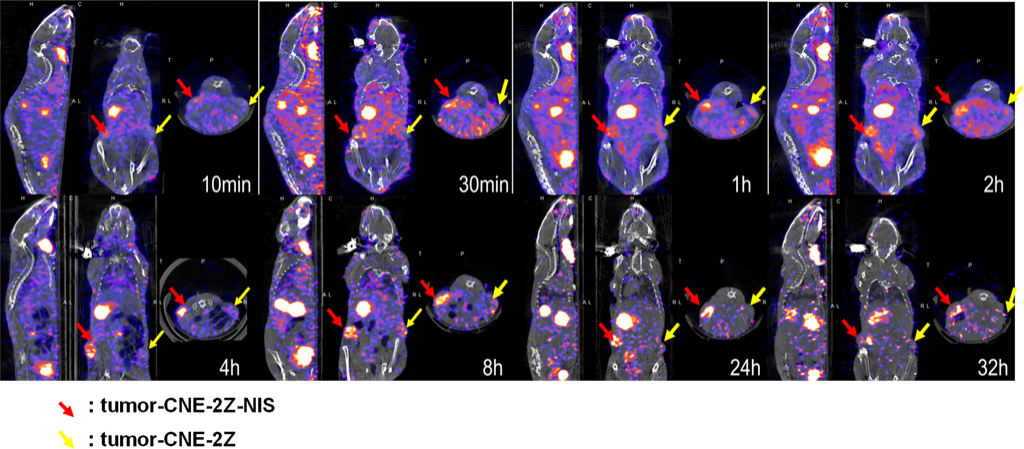
Dynamic ^125^I SPECT/CT imaging *in vivo*. Micro-SPECT/CT imaging of mice bearing CNE-2Z tumors (yellow arrow) and CNE-2Z-NIS tumors (red arrow) after injection of ^125^I (10.5 MBq) for 10min–32h.

Significant radioactive uptake was observed by the CNE-2Z-NIS tumors from 30 min to 8 h, peaking within 4 h; however, ^125^I uptake was not detectable in CNE-2Z tumors. The radioactivity of CNE-2Z-NIS tumors slightly decreased after 8 h; however, radioactivity could still be detected 32 h after injection of ^125^I. (Fig. 4).

**Figure 5:**
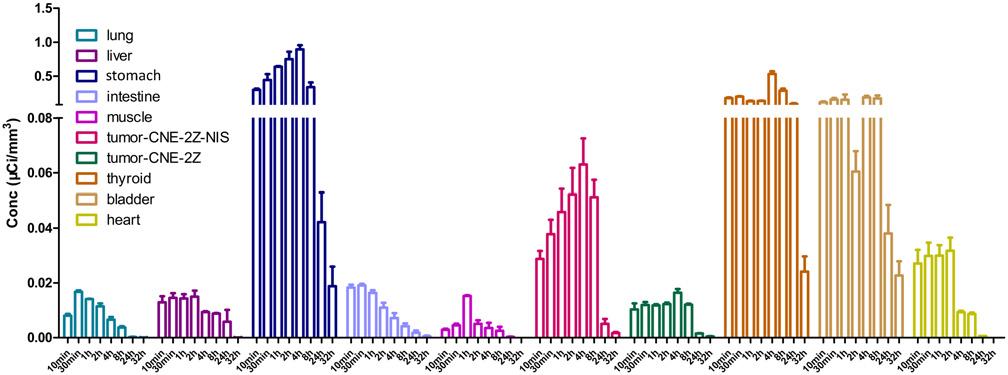
Quantitative analysis of ^125^I uptake *in vivo*. Conc values (μCi/mm^3^) of the xenograft tumors and organs after injection of ^125^I (same time points as in Fig. 4).

ROIs were created by CT positioning during SPECT imaging to define the tissues described above and the Conc values were obtained at various time points. Accumulation of ^125^I increased gradually in CNE-2Z-NIS tumors and reached the highest Conc value at 4 h after injection of ^125^I; this was significantly (up to 75%) higher than the corresponding values for CNE-2Z tumors. And then decreased by 8 h and reached the lowest Conc value at 32 h. Significant radioiodine accumulation was also observed in tissues which express endogenous *NIS*, including the thyroid and stomach, and also in the urinary bladder and heart due to renal elimination and absorption of the radionuclide into the bloodstream. The Conc values of the lung, liver, muscle and the intestine remained low at all time points (Fig. 5).

### ^131^I reduces the survival of NPC cells expressing NIS *in vitro*

**Figure 6:**
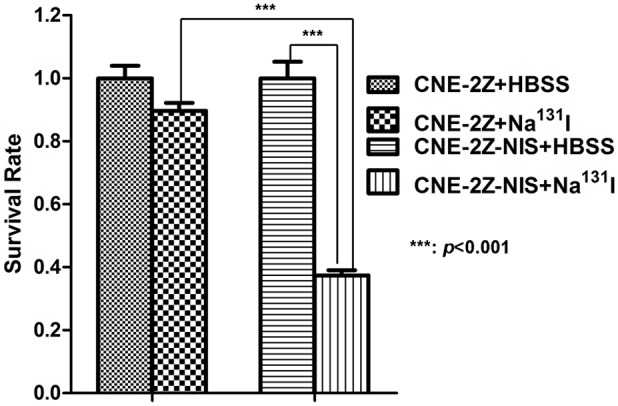
Cytotoxic effect of ^131^I towards CNE-2Z-NIS cells *in vitro*. CNE-2Z and CNE-2Z-NIS cells (6 × 10^6^ cells/dish) were incubated with 4.6 MBq ^131^I in HBSS for 8 h. Subsequently, cells were washed with HBSS, trypsinized and 1,000 cells were plated into each well of 6-well culture plates for 7 days. The survival rate was expressed as the percentage of colonies to that of the blank group without ^131^I incubation. Data are presented as means ± SD.

*In vitro* clonogenic assays were performed to determine the effect of ^131^I in CNE-2Z-NIS and CNE-2Z cells. ^131^I had a significant cytotoxic effect in CNE-2Z-NIS cells compared to CNE-2Z cells (*p* < 0.001) and control CNE-2Z-NIS cells treated with HBSS (*p* < 0.001) (Fig. 6).

### Therapeutic effects of ^131^I in NPC xenograft tumors expressing NIS *in vivo*

**Figure 7:**
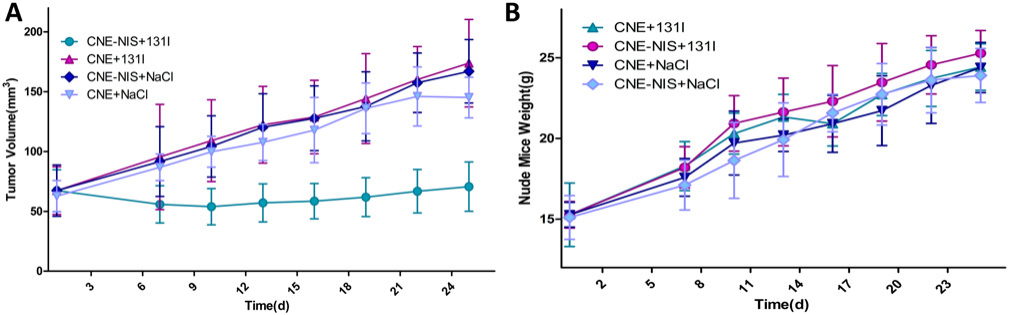
Therapeutic effects of ^131^I on CNE-2Z-NIS xenografts. (A) During the 25 day period after injection of ^131^I or PBS, the growth of CNE-2Z-NIS and CNE-2Z xenografts were measured at different time. Data are present as means ± SD. (B) Weight of the mice during the 25 day period after injection of ^131^I. Data are present as means ± SD.

**Figure 8:**
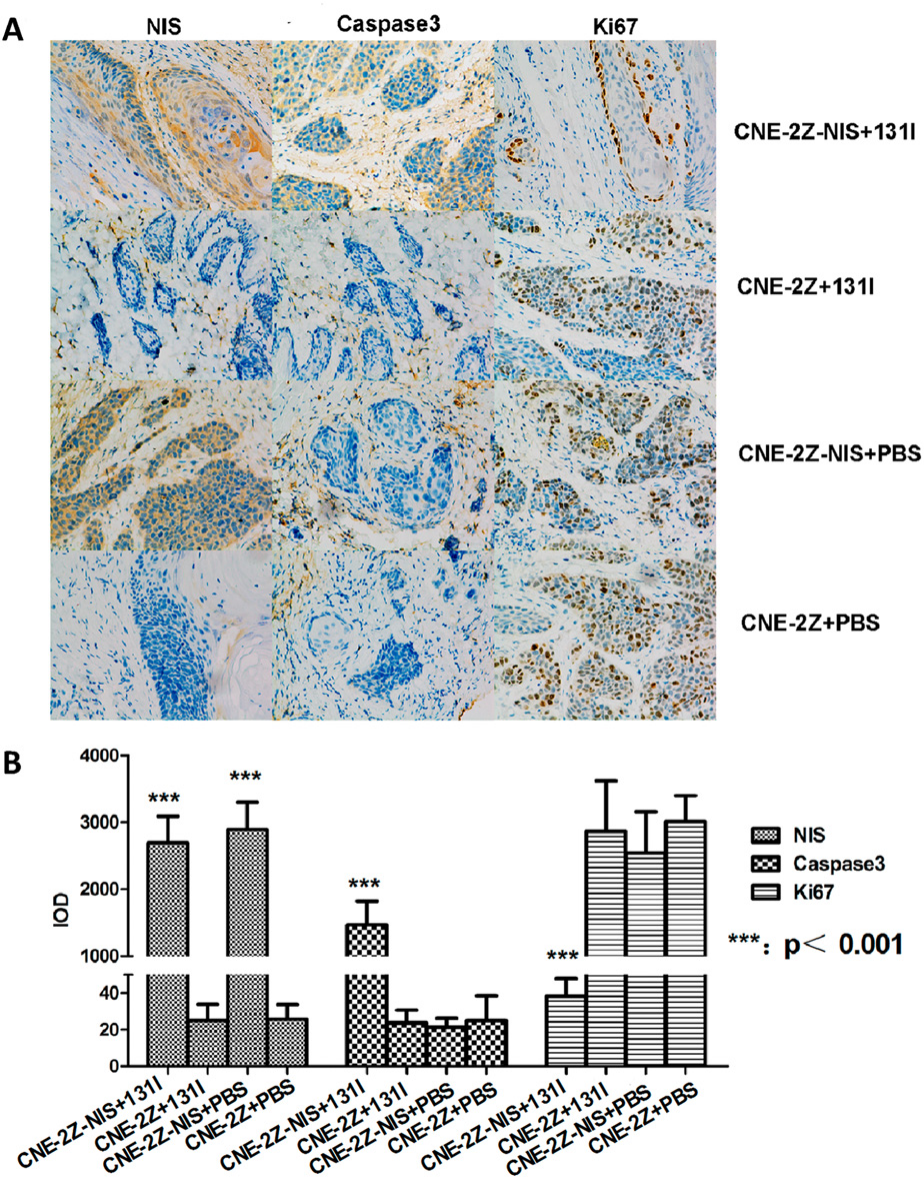
Immunohistochemical staining. (A) Expression of NIS, caspase-3 and Ki-67 in CNE-2Z and CNE-2Z-NIS xenograft tumors treated with ^131^I or PBS (magnification, 200×). (B) Immunohistochemical analysis of protein NIS, caspase3 and Ki67. High expression of NIS protein was observed in the cells of the CNE-2Z-NIS xenografts with ^131^I or PBS treatment compared with the cells of the CNE-2Z xenografts with ^131^I or PBS treatment (*p*<0.001). High expression of Caspase3 protein was observed in the cells of the CNE-2Z-NIS xenografts treated with ^131^I compared with the other three groups (*p*<0.001), but low expression of Ki67 protein in the cells of the CNE-2Z-NIS xenografts treated with ^131^I was observed compared with the other three groups (*p*<0.001). Data are present as means ± SD.

Therapy with ^131^I was initiated when the tumors reached 3–5 mm in diameter (~70mm^3^). ^131^I significantly inhibited the growth of CNE-2Z-NIS tumors compared to CNE-2Z tumors treated with ^131^I, CNE-2Z-NIS and CNE-2Z tumors treated with PBS (P<0.001). There is no significant difference among the growth of CNE-2Z tumors treated with ^131^I, CNE-2Z-NIS and CNE-2Z tumors treated with PBS (P>0.05) (Fig. 7A). Therapy did not affect food intake or physical activity. During treatment, the weight of the CNE-2Z-NIS tumor-bearing mice treated with ^131^I increased at almost the same rate as the CNE-2Z tumor-bearing mice treated with ^131^I, CNE-2Z-NIS and CNE-2Z tumor-bearing mice treated with PBS (P>0.05) (Fig. 7B). Representative images of the indirect immunohistochemical staining of the tumor xenografts are shown in Fig. 8A. Immunohistochemical analysis shows that high expression of NIS protein was observed in the cells of the CNE-2Z-NIS xenografts with ^131^I or PBS treatment compared with the cells of the CNE-2Z xenografts with ^131^I or PBS treatment (*p*<0.001, Fig. 8B). Higher expression of Caspase3 protein was observed in the cells of CNE-2Z-NIS xenografts treated with ^131^I compared with the cells of the CNE-2Z xenografts treated with ^131^I, CNE-2Z-NIS xenografts treated with PBS and CNE-2Z xenografts treated with PBS (*p*<0.001, Fig. 8B), but lower expression of Ki67 protein in the cells of the CNE-2Z-NIS xenografts treated with ^131^I was observed compared with the other three groups (*p*<0.001, Fig. 8B).

## Discussion

As many patients with NPC experience recurrent disease after undergoing current conventional treatment methods, novel efficient therapies need to be explored. The development of new therapies, such as targeted gene therapies may provide an effective and non-toxic method of treating NPC. Gene therapy strategies based on p53 [15–17], p16 [18] and FasL [19] have demonstrated therapeutic potential in NPC. A large number of viruses have been employed for gene therapy in animal studies and clinical trials, including adenoviruses, adeno-associated viruses, herpes viruses, poxviruses and retroviruses. In recent years, research has focused on the use of lentiviruses. Third generation lentiviral packaging systems possess a number of advantages: the packaging plasmids only encode the essential proteins required for lentivector assembly and function [20–21], and the vectors can accommodate large transgenes [up to ~10 kilobases (kb)] [22], can potentially be used for *ex vivo* or *in vivo* gene transfer into dividing and non-dividing cells [23]. However, lentivirus have several disadvantages, one of the most relevant being that the transgenes are easily integrated into the genomic DNA of host cells and thus are potentially dangerous [24]. For this reason, baculoviruses have been introduced as more safe viral vectors, they neither replicating in mammalian cells nor randomly integrating into the host genome [25, 26].

In previous studies, *NIS* was effectively applied as a therapeutic gene to enhance the uptake of ^131^I, ^188^Re or ^211^At [11, 27, 28]. In this study, we successfully constructed a cell line expressing the *NIS* and *EGFP* genes using a lentivector driven by a single promoter, EF1α. The *NIS* gene could be used for molecular imaging and gene-mediated radioisotope therapy, while the EGFP gene facilitated FACS analysis and cell sorting *in vitro*.

To our knowledge, our study is the first to use a lentivirus to deliver the *NIS* gene for the treatment of NPC tumor xenografts *in vivo*. The Lv-EF1α-NIS-IRES-EGFP lentiviral particles were successfully packaged and could efficiently infect CNE-2Z cells. CNE-2Z cells stably expressing NIS and EGFP (termed CNE-2Z-NIS cells) were sorted by flow cytometry, and the expression of NIS was confirmed using qPCR, immunofluorescence and Western blotting. To address concerns regarding the biosafety of the lentivirus, we assessed the effects of exogenous NIS expression on the viability and proliferation of CNE-2Z cells *in vitro*; however, no significant differences in the cell viability or proliferation of CNE-2Z-NIS and CNE-2Z cells were observed.

For effective treatment, it is vital that the tumor receives and maintains a high dose of the radioisotope over a long period of time. The dose of ^131^I received by cells expressing NIS is determined by the levels of NIS expressed, the dose of the radioisotope, and the effective half-life of the isotope in the tumor, which is determined by the physical half-life (8.021 days for ^131^I) and biological half-life of the isotope. In our *in vivo* experiments, significant ^125^I uptake was observed in the CNE-2Z-NIS xenografts from 30 min to 8 h after injection of ^125^I, reaching maximal levels within 4 h. The accumulation of ^125^I in CNE-2Z-NIS tumors increased gradually and reached the highest Conc value 4 h after injection of ^125^I, and was significantly (up to 75%) higher than the accumulation of ^125^I in CNE-2Z xenografts. In summary, the lentivirus generated long-lasting, stable expression of NIS which enabled high, sustained levels of ^125^I uptake and accumulation in the CNE-2Z-NIS xenografts for at least 8 h after injection of ^125^I; these results are superior to a previous study in which human stem cells were transiently transfected with *NIS* using baculoviruses [29].

We also investigated the therapeutic effects of ^131^I radionuclide therapy. Clonogenic assays showed that ^131^I efficiently and specifically killed CNE-2Z-NIS tumor cells *in vitro* (compared to CNE-2Z cells and control CNE-2Z-NIS cells treated with HBSS). In the tumor xenograft model, ^131^I treatment significantly retarded the growth of CNE-2Z-NIS tumors whereas the CNE-2Z tumors treated with ^131^I, CNE-2Z-NIS and CNE-2Z tumors treated with PBS continued increase in volume. In addition, immunohistochemical analysis for Ki67 and caspase-3 showed significantly lower numbers of proliferating cells and increased levels of apoptosis after treatment with ^131^I in CNE-2Z-NIS tumors compared to CNE-2Z tumors treated with ^131^I, CNE-2Z-NIS and CNE-2Z tumors treated with PBS, suggesting that ^131^I uptake by CNE-2Z-NIS cells induced tumor cell damage in addition to tumor cell death.

Implantation of ^125^I radioactive seeds into NPC tumor tissues, under the guidance of positron emission tomography combined with computed tomography (PET-CT), proved to be an acceptable and feasible treatment with minimal damage and few complications in refractory NPC [30]. ^131^I has a half-life of 8 days, and expends 971 KeV of decay energy with gamma decay following rapidly after beta decay, which is more powerful than ^125^I. The electrons have a tissue penetration of 0.6 to 2 mm [31], which induces a low degree of injury to the healthy tissues around the tumor. This indicates that the use of NIS gene therapy in combination with ^131^I may have significant potential as an effective, low toxicity treatment in NPC. In our study, ^131^I treatment significantly retarded the growth of CNE-2Z-NIS xenografts but did not affect mice’s food intake or physical activity.

The *NIS* gene must be properly targeted to the tumor cell membrane to be functional [32]; however, the EF1α promoter can drive the expression of transgenes in a wide variety of human cell lines and has no tumor-specificity [33, 34]. The use of cell-specific promoters *in vivo* is advantageous due to their lower sensitivity to promoter inactivation and lower risk of activating the host cell defense machinery [35]. As such, future studies will focus on exploring and testing novel gene promoters that specifically target NPC cells to achieve safer, more specific, robust and stable therapeutic effects for radionuclide therapy in NPC, and the use of more powerful therapeutic radionucleotides, such as ^211^At or ^188^Re, should be investigated in NPC cells expressing *NIS*.

## Conclusion

EF1α promoter-driven expression of *NIS* enabled significant uptake and accumulation of radioisotopes in NPC cells, and provided effective therapeutic effects *in vitro* and *in vivo*. NIS gene therapy in combination with radionuclide treatment deserves further research as a novel treatment for NPC.

## References

[1] Wei WI, Sham JS (2005) Nasopharyngeal carcinoma. Lancet 365: 2041–2054. 10.1016/S0140-6736(05)66698-6 15950718

[2] Xu T, Tang J, Gu M, Liu L, Wei W, et al (2013) Recurrent nasopharyngeal carcinoma: a clinical dilemma and challenge. Curr Oncol 20: e406–419. 10.3747/co.20.1456 24155638 PMC3805410

[3] Li JX, Lu TX, Huang Y, Han F, Chen CY, et al (2010) [Clinical features of 337 patients with recurrent nasopharyngeal carcinoma]. Chin J Cancer 29: 82–86. 10.5732/cjc.009.10412 20038316

[4] Li X, Liu X, Li CY, Ding Y, Chau D, et al (2006) Recombinant adeno-associated virus mediated RNA interference inhibits metastasis of nasopharyngeal cancer cells in vivo and in vitro by suppression of Epstein-Barr virus encoded LMP-1. Int J Oncol 29: 595–603. 16865275

[5] Dai G, Levy O, Carrasco N (1996) Cloning and characterization of the thyroid iodide transporter. Nature 379: 458–460. 10.1038/379458a0 8559252

[6] Bosch EH, van Doorne H, de Vries S (2000) The lactoperoxidase system: the influence of iodide and the chemical and antimicrobial stability over the period of about 18 months. J Appl Microbiol 89: 215–224. 10.1046/j.1365-2672.2000.01098.x 10971753

[7] Mazzaferri EL, Kloos RT (2001) Clinical review 128: Current approaches to primary therapy for papillary and follicular thyroid cancer. J Clin Endocrinol Metab 86: 1447–1463. 10.1210/jcem.86.4.7407 11297567

[8] Riesco-Eizaguirre G, Santisteban P (2006) A perspective view of sodium iodide symporter research and its clinical implications. Eur J Endocrinol 155: 495–512. 10.1530/eje.1.02257 16990649

[9] Hingorani M, Spitzweg C, Vassaux G, Newbold K, Melcher A, et al (2010) The biology of the sodium iodide symporter and its potential for targeted gene delivery. Curr Cancer Drug Targets 10: 242–267. 10.2174/156800910791054194 20201784 PMC3916908

[10] Vannucci L, Lai M, Chiuppesi F, Ceccherini-Nelli L, Pistello M (2013) Viral vectors: a look back and ahead on gene transfer technology. New Microbiol 36: 1–22. 23435812

[11] Guo R, Zhang R, Pan Y, Xu H, Zhang M, et al (2011) Feasibility of a novel positive feedback effect of 131I-promoted Bac-Egr1-hNIS expression in malignant glioma through baculovirus: a comparative study with Bac-CMV-hNIS. Nucl Med Commun 32: 402–409. 10.1097/MNM.0b013e328344a1ad 21386735

[12] Livak KJ, Schmittgen TD (2001) Analysis of relative gene expression data using real-time quantitative PCR and the 2(-Delta Delta C(T)) Method. Methods 25: 402–408. 10.1006/meth.2001.1262 11846609

[13] Weiss SJ, Philp NJ, Grollman EF (1984) Iodide transport in a continuous line of cultured cells from rat thyroid. Endocrinology 114: 1090–1098. 10.1210/endo-114-4-1090 6705729

[14] Sides MD, Sosulski ML, Luo F, Lin Z, Flemington EK, et al (2013) Co-treatment with arsenic trioxide and ganciclovir reduces tumor volume in a murine xenograft model of nasopharyngeal carcinoma. Virol J 10: 152 10.1186/1743-422X-10-152 23680002 PMC3666899

[15] Li JH, Li P, Klamut H, Liu FF (1997) Cytotoxic effects of Ad5CMV-p53 expression in two human nasopharyngeal carcinoma cell lines. Clin Cancer Res 3: 507–514. 9815713

[16] Li JH, Huang D, Sun BF, Zhang X, Middeldorp J, et al (2000) Efficacy of ionizing radiation combined with adenoviral p53 therapy in EBV-positive nasopharyngeal carcinoma. Int J Cancer 87: 606–610. 10.1002/1097-0215(20000815)87:4<606::AID-IJC23>3.0.CO;2-O 10918205

[17] Weinrib L, Li JH, Donovan J, Huang D, Liu FF (2001) Cisplatin chemotherapy plus adenoviral p53 gene therapy in EBV-positive and -negative nasopharyngeal carcinoma. Cancer Gene Ther 8: 352–360. 10.1038/sj.cgt.7700319 11477455

[18] Wang GL, Lo KW, Tsang KS, Chung NY, Tsang YS, et al (1999) Inhibiting tumorigenic potential by restoration of p16 in nasopharyngeal carcinoma. Br J Cancer 81: 1122–1126. 10.1038/sj.bjc.6690818 10584871 PMC2374319

[19] Li JH, Shi W, Chia M, Sanchez-Sweatman O, Siatskas C, et al (2003) Efficacy of targeted FasL in nasopharyngeal carcinoma. Mol Ther 8: 964–973. 10.1016/j.ymthe.2003.08.018 14664799

[20] Vigna E, Naldini L (2000) Lentiviral vectors: excellent tools for experimental gene transfer and promising candidates for gene therapy. J Gene Med 2: 308–316. 10.1002/1521-2254(200009/10)2:5<308::AID-JGM131>3.0.CO;2-3 11045424

[21] Cockrell AS, Kafri T (2007) Gene delivery by lentivirus vectors. Mol Biotechnol 36: 184–204. 10.1007/s12033-007-0010-8 17873406

[22] De Meyer SF, Vanhoorelbeke K, Chuah MK, Pareyn I, Gillijns V, et al (2006) Phenotypic correction of von Willebrand disease type 3 blood-derived endothelial cells with lentiviral vectors expressing von Willebrand factor. Blood 107: 4728–4736. 10.1182/blood-2005-09-3605 16478886 PMC1895808

[23] Naldini L, Blomer U, Gallay P, Ory D, Mulligan R, et al (1996) In vivo gene delivery and stable transduction of nondividing cells by a lentiviral vector. Science 272: 263–267. 10.1126/science.272.5259.263 8602510

[24] Moiani A, Paleari Y, Sartori D, Mezzadra R, Miccio A, et al (2012) Lentiviral vector integration in the human genome induces alternative splicing and generates aberrant transcripts. J Clin Invest 122(5): 1653–1666. 10.1172/JCI61852 22523069 PMC3347495

[25] Hu YC (2008) Baculoviral vectors for gene delivery: A review. Curr Gene Ther 8: 54–65. 10.2174/156652308783688509 18336250

[26] Zhang XJ, Godbey WT (2006) Viral vectors for gene delivery in tissue engineering. Adv Drug Deliv Rev 58: 515–534. 10.1016/j.addr.2006.03.006 16762441

[27] Petrich T, Quintanilla-Martinez L, Korkmaz Z, Samson E, Helmeke HJ, et al (2006) Effective cancer therapy with the alpha-particle emitter [211At]astatine in a mouse model of genetically modified sodium/iodide symporter-expressing tumors. Clin Cancer Res 12: 1342–1348. 10.1158/1078-0432.CCR-05-1576 16489092

[28] Weeks AJ, Jauregui-Osoro M, Cleij M, Blower JE, Ballinger JR, et al (2011) Evaluation of [18F]-tetrafluoroborate as a potential PET imaging agent for the human sodium/iodide symporter in a new colon carcinoma cell line, HCT116, expressing hNIS. Nucl Med Commun 32(2): 98–105. 10.1097/MNM.0b013e3283419540 21085047 PMC6219699

[29] Pan Y, Liu S, Wu H, Lv J, Xu X, et al (2013) Baculovirus as an ideal radionuclide reporter gene vector: a new strategy for monitoring the fate of human stem cells in vivo. PLoS One 8: e61305 10.1371/journal.pone.0061305 23596521 PMC3626603

[30] Zhang F, Wu K, Gao F, Zhang W, Shi F, et al (2013) Refractory nasopharyngeal carcinoma: positron emission tomography combined with computed tomography-guided 125I seed implantation therapy after repeated traditional radiochemotherapy. Otolaryngol Head Neck Surg 149: 417–423. 10.1177/0194599813491221 23715683

[31] Skugor M (2006) The Cleveland Clinic Guide to Thyroid Disorders. New York: Kaplan Publisher 82 p.

[32] Kaminsky SM, Levy O, Salvador C, Dai G, Carrasco N (1993) The Na+/I- symporter of the thyroid gland. Soc Gen Physiol Ser 48: 251–262. 8503049

[33] Almeciga-Diaz CJ, Rueda-Paramo MA, Espejo AJ, Echeverri OY, Montano A, et al (2009) Effect of elongation factor 1alpha promoter and SUMF1 over in vitro expression of N-acetylgalactosamine-6-sulfate sulfatase. Mol Biol Rep 36: 1863–1870. 10.1007/s11033-008-9392-3 18989752

[34] Qin JY, Zhang L, Clift KL, Hulur I, Xiang AP, et al (2010) Systematic comparison of constitutive promoters and the doxycycline-inducible promoter. PLoS One 5: e10611 10.1371/journal.pone.0010611 20485554 PMC2868906

[35] Liu BH, Wang X, Ma YX, Wang S (2004) CMV enhancer/human PDGF-beta promoter for neuron-specific transgene expression. Gene Ther 11: 52–60. 10.1038/sj.gt.3302126 14681697

